# Bis{μ-4′-[4-(quinolin-8-yloxymeth­yl)phen­yl]-2,2′:6′,2′′-terpyridine}disilver(I) bis­(perchlorate) dimethyl­formamide disolvate

**DOI:** 10.1107/S1600536809046832

**Published:** 2009-11-25

**Authors:** Chang-Juan Chen, Feng-Neng Liu, Ai-Jiang Zhang, Liang-Wei Zhang, Xiang Liu

**Affiliations:** a State Key Laboratory of Applied Organic Chemistry, College of Chemistry and Chemical Engineering, Lanzhou University, Lanzhou 730000, People’s Republic of China

## Abstract

In the binuclear title complex, [Ag_2_(C_31_H_22_N_4_O)_2_](ClO_4_)_2_·2C_3_H_7_NO, the Ag^I^ atom is penta­coordinated by three N atoms from the tridentate chelating terpyridyl group and by one N atom and one O atom from the quinolin-8-yl­oxy group in a distorted square-pyramidal geometry with the O atom at the apical position. The centrosymmetric complex cation involves intra­molecular π–π stacking inter­actions [centroid–centroid distance = 3.862 (4) Å] between the central pyridine and benzene rings. In the crystal structure, inter­molecular C—H⋯O hydrogen bonds result in the formation of a supra­molecular network.

## Related literature

For applications of 2,2′:6′,2′′-terpyridine in supra­molecular frameworks and functional materials, see: Andres & Schubert (2004 ▶); Constable *et al.* (2005 ▶); Thompson (1997 ▶); Ziener *et al.* (2000 ▶). For the ligand synthesis, see: Chow *et al.* (2006 ▶). For related structures, see: Hou & Li (2005 ▶).
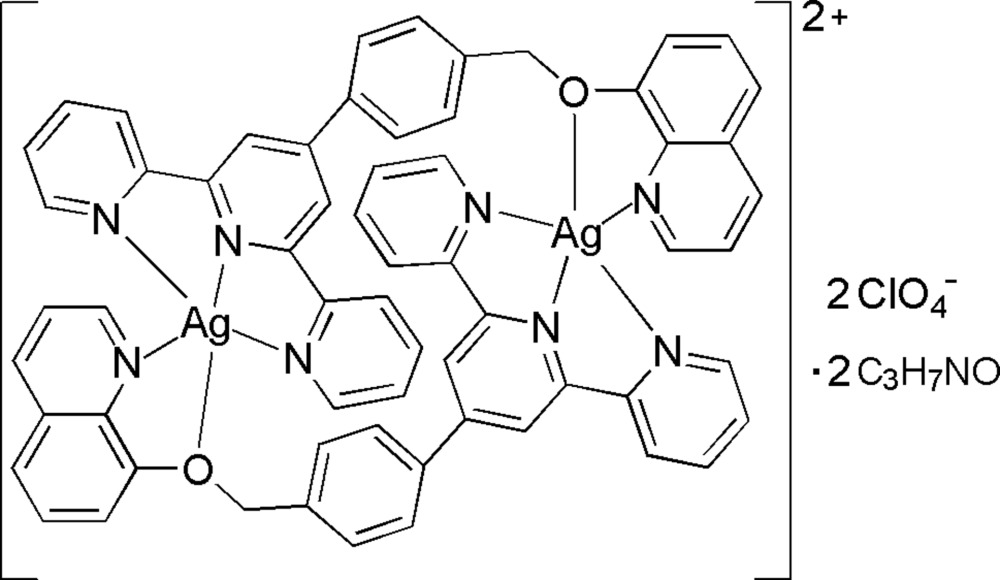


## Experimental

### 

#### Crystal data

[Ag_2_(C_31_H_22_N_4_O)_2_](ClO_4_)_2_·2C_3_H_7_NO
*M* *_r_* = 1493.88
Monoclinic, 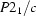

*a* = 10.0552 (16) Å
*b* = 10.9548 (18) Å
*c* = 29.657 (5) Å
β = 96.767 (8)°
*V* = 3244.0 (9) Å^3^
*Z* = 2
Mo *K*α radiation
μ = 0.76 mm^−1^
*T* = 296 K
0.22 × 0.20 × 0.20 mm

#### Data collection

Bruker APEXII CCD diffractometer
Absorption correction: multi-scan (*SADABS*; Sheldrick, 1996 ▶) *T*_min_ = 0.847, *T*_max_ = 0.860
20993 measured reflections
8077 independent reflections
5406 reflections with *I* > 2σ(*I*)
*R*_int_ = 0.020

#### Refinement

*R*[*F* ^2^ > 2σ(*F* ^2^)] = 0.041
*wR*(*F* ^2^) = 0.118
*S* = 1.03
8077 reflections
426 parameters
H-atom parameters constrained
Δρ_max_ = 0.67 e Å^−3^
Δρ_min_ = −0.44 e Å^−3^

### 

Data collection: *APEX2* (Bruker, 2007 ▶); cell refinement: *SAINT* (Bruker, 2007 ▶); data reduction: *SAINT*; program(s) used to solve structure: *SHELXS97* (Sheldrick, 2008 ▶); program(s) used to refine structure: *SHELXL97* (Sheldrick, 2008 ▶); molecular graphics: *SHELXTL* (Sheldrick, 2008 ▶); software used to prepare material for publication: *SHELXTL*.

## Supplementary Material

Crystal structure: contains datablocks I, global. DOI: 10.1107/S1600536809046832/hy2238sup1.cif

Structure factors: contains datablocks I. DOI: 10.1107/S1600536809046832/hy2238Isup2.hkl

Additional supplementary materials:  crystallographic information; 3D view; checkCIF report

## Figures and Tables

**Table 1:** Selected bond lengths (Å)

Ag1—O1	2.5995 (19)
Ag1—N1	2.434 (3)
Ag1—N2	2.3572 (19)
Ag1—N3	2.414 (3)
Ag1—N4	2.275 (2)

**Table 2:** Hydrogen-bond geometry (Å, °)

*D*—H⋯*A*	*D*—H	H⋯*A*	*D*⋯*A*	*D*—H⋯*A*
C4—H4⋯O3^i^	0.93	2.57	3.450 (5)	158
C12—H12⋯O5^ii^	0.93	2.60	3.344 (5)	138
C28—H28⋯O6^iii^	0.93	2.37	3.250 (5)	158
C29—H29⋯O4	0.93	2.57	3.252 (6)	130

Symmetry codes: (i) 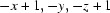; (ii) 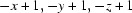; (iii) 
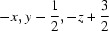
.

**Fig. 1.**
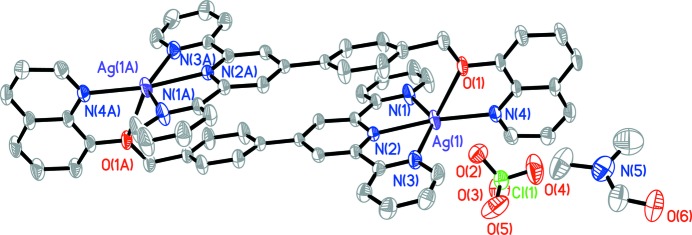
Molecular structure of the title compound. Displacement ellipsoids are drawn at the 30% probability level. H atoms have been omitted for clarity. [Symmetry code: (A) 2 - z, 1 - y, 1 - z.]

**Fig. 2.**
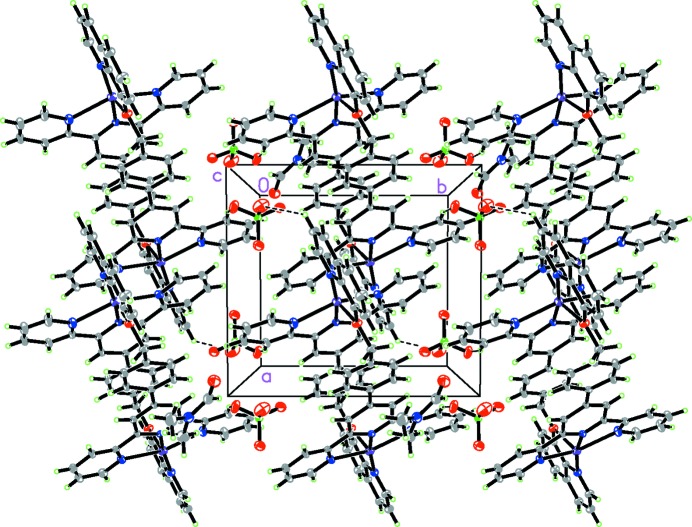
Packing diagram along the c axis of the title compound. Hydrogen bonds are shown as dashed lines.

## supplementary crystallographic information

### Comment

2,2':6',2''-Terpyridine is well known for its applications in the synthesis of supramolecular frameworks and functional materials (Andres & Schubert, 2004; Constable *et al.*, 2005; Thompson, 1997; Ziener *et al.*, 2000). Therefore, it is necessary to further widen the system of multifunctional terpyridyl derivates complexes. Herein we report the synthesis and structure of an Ag^I^ complex with a new ligand 4'-[4'-(8-oxyqulinoline)benzyl]-2,2':6',2''-terpyridine.

The complex cation of the title compound has a twisted box structure formed by two ligands bridging two Ag^I^ atoms. The asymmetric unit consists of a half of the cation, one perchlorate anion and one dimethylformamide solvent molecule. As shown Fig. 1, the two ligands are arranged in a head-to-tail fashion. The Ag^I^ atom is pentacoordinated by three N atoms of the tridentate chelating terpyridyl group and by one N and one O atoms from the quinolin-8-yloxy group [Ag—N = 2.275 (2)–2.434 (3) Å and Ag—O = 2.5995 (19) Å] (Table 1). The Ag···Ag distance is 10.744 (6) Å in the dimeric complex cation. The plane of the 4'-phenyl-2,2':6',2''-terpyridine group is almost perpendicular to that of the quinoline group, with a dihedral angel of 79.96 (1)°. The complex cation involves π–π stacking interactions [centroid–centroid distance = 3.862 (4) Å] between the central pyridine rings and the phenyl rings. In the crystal structure, intermolecular C—H···O hydrogen bonds result in the formation of a supramolecular network (Table 2 and Fig. 2).

### Experimental

The ligand was synthesized according to the literature (Chow *et al.*, 2006). A solution of AgClO_4_(23.72 mg, 0.10 mmol) in CH_3_OH (10 ml) was added to a solution of the ligand (46.6 mg, 0.10 mmol) in CHCl_3_ (10 ml) with stirring. The brown precipitate was collected by filtration and washed sequentially with 10 ml CH_3_OH and CHCl_3_. Brown crystals suitable for X-ray structure determination were obtained by slow diffusion of diethyl ether into the DMF solution of the product in several days.

### Refinement

H atoms were positioned geometrically and refined as riding atoms, with C—H = 0.93 (aromatic), 0.97 (CH_2_) and 0.96 (CH_3_) Å and with *U*_iso_ = 1.2(1.5 for methyl)*U*_eq_(C).

### Crystal data

**Table:**

[Ag_2_(C_31_H_22_N_4_O)_2_](ClO_4_)_2_·2C_3_H_7_NO	*F*(000) = 1520
*M**_r_* = 1493.88	*D*_x_ = 1.529 Mg m^−^^3^
Monoclinic, *P*2_1_/*c*	Mo *K*α radiation, λ = 0.71073 Å
Hall symbol: -P 2ybc	Cell parameters from 6845 reflections
*a* = 10.0552 (16) Å	θ = 2.3–28.6°
*b* = 10.9548 (18) Å	µ = 0.76 mm^−^^1^
*c* = 29.657 (5) Å	*T* = 296 K
β = 96.767 (8)°	Block, yellow
*V* = 3244.0 (9) Å^3^	0.22 × 0.20 × 0.20 mm
*Z* = 2	

### Data collection

**Table:**

Bruker APEXII CCD diffractometer	8077 independent reflections
Radiation source: fine-focus sealed tube	5406 reflections with *I* > 2σ(*I*)
graphite	*R*_int_ = 0.020
φ and ω scans	θ_max_ = 28.7°, θ_min_ = 2.0°
Absorption correction: multi-scan (*SADABS*; Sheldrick, 1996)	*h* = −12→12
*T*_min_ = 0.847, *T*_max_ = 0.860	*k* = −14→14
20993 measured reflections	*l* = −40→28

### Refinement

**Table:**

Refinement on *F*^2^	Primary atom site location: structure-invariant direct methods
Least-squares matrix: full	Secondary atom site location: difference Fourier map
*R*[*F*^2^ > 2σ(*F*^2^)] = 0.041	Hydrogen site location: inferred from neighbouring sites
*wR*(*F*^2^) = 0.118	H-atom parameters constrained
*S* = 1.03	*w* = 1/[σ^2^(*F*_o_^2^) + (0.0551*P*)^2^ + 1.1422*P*] where *P* = (*F*_o_^2^ + 2*F*_c_^2^)/3
8077 reflections	(Δ/σ)_max_ = 0.001
426 parameters	Δρ_max_ = 0.67 e Å^−^^3^
0 restraints	Δρ_min_ = −0.44 e Å^−^^3^

### Fractional atomic coordinates and isotropic or equivalent isotropic displacement parameters (Å^2^)

**Table:**

	*x*	*y*	*z*	*U*_iso_*/*U*_eq_	
Ag1	0.60078 (2)	0.42357 (2)	0.602143 (7)	0.06913 (11)	
C1	0.6898 (5)	0.1295 (4)	0.61912 (13)	0.1029 (14)	
H1	0.6456	0.1402	0.6447	0.123*	
C2	0.7329 (5)	0.0155 (4)	0.60987 (14)	0.1074 (15)	
H2	0.7210	−0.0493	0.6293	0.129*	
C3	0.7939 (4)	−0.0024 (3)	0.57173 (14)	0.0904 (12)	
H3	0.8235	−0.0795	0.5645	0.108*	
C4	0.8109 (3)	0.0965 (3)	0.54406 (11)	0.0698 (9)	
H4	0.8512	0.0863	0.5176	0.084*	
C5	0.7678 (3)	0.2102 (2)	0.55580 (9)	0.0534 (6)	
C6	0.7810 (2)	0.3201 (2)	0.52706 (8)	0.0463 (5)	
C7	0.8646 (2)	0.3213 (2)	0.49286 (8)	0.0487 (6)	
H7	0.9159	0.2530	0.4880	0.058*	
C8	0.8716 (2)	0.4248 (2)	0.46597 (7)	0.0412 (5)	
C9	0.7944 (2)	0.5249 (2)	0.47579 (8)	0.0459 (5)	
H9	0.7971	0.5964	0.4590	0.055*	
C10	0.7135 (2)	0.5180 (2)	0.51051 (8)	0.0432 (5)	
C11	0.6290 (2)	0.6236 (2)	0.52240 (8)	0.0472 (6)	
C12	0.6129 (3)	0.7268 (3)	0.49576 (11)	0.0631 (7)	
H12	0.6549	0.7329	0.4695	0.076*	
C13	0.5341 (3)	0.8211 (3)	0.50828 (13)	0.0785 (9)	
H13	0.5232	0.8918	0.4909	0.094*	
C14	0.4719 (3)	0.8092 (3)	0.54682 (13)	0.0810 (10)	
H14	0.4186	0.8714	0.5563	0.097*	
C15	0.4906 (3)	0.7035 (3)	0.57070 (12)	0.0805 (10)	
H15	0.4477	0.6950	0.5966	0.097*	
C16	0.9562 (2)	0.4271 (2)	0.42791 (8)	0.0442 (5)	
C17	1.0168 (4)	0.3227 (3)	0.41471 (11)	0.0782 (10)	
H17	1.0084	0.2507	0.4307	0.094*	
C18	1.0903 (4)	0.3227 (3)	0.37802 (12)	0.0806 (10)	
H18	1.1317	0.2510	0.3703	0.097*	
C19	1.1035 (3)	0.4254 (3)	0.35296 (9)	0.0544 (6)	
C20	1.0478 (3)	0.5310 (3)	0.36688 (10)	0.0680 (8)	
H20	1.0580	0.6030	0.3510	0.082*	
C21	0.9763 (3)	0.5325 (3)	0.40425 (10)	0.0601 (7)	
H21	0.9415	0.6059	0.4134	0.072*	
C22	1.1736 (3)	0.4222 (3)	0.31074 (10)	0.0654 (8)	
H22A	1.1898	0.3384	0.3023	0.078*	
H22B	1.1186	0.4611	0.2857	0.078*	
C23	0.6229 (3)	0.5043 (3)	0.71396 (8)	0.0558 (7)	
C24	0.6558 (4)	0.5487 (3)	0.75690 (10)	0.0733 (9)	
H24	0.7364	0.5895	0.7645	0.088*	
C25	0.5659 (4)	0.5321 (4)	0.78984 (11)	0.0891 (11)	
H25	0.5892	0.5605	0.8192	0.107*	
C26	0.4473 (4)	0.4757 (4)	0.77919 (11)	0.0804 (10)	
H26	0.3892	0.4669	0.8012	0.097*	
C27	0.4105 (3)	0.4299 (3)	0.73521 (10)	0.0609 (7)	
C28	0.2875 (3)	0.3705 (3)	0.72234 (11)	0.0717 (9)	
H28	0.2256	0.3621	0.7431	0.086*	
C29	0.2598 (3)	0.3259 (3)	0.67989 (12)	0.0744 (9)	
H29	0.1796	0.2853	0.6713	0.089*	
C30	0.3534 (3)	0.3414 (3)	0.64886 (10)	0.0710 (8)	
H30	0.3333	0.3094	0.6198	0.085*	
C31	0.4993 (3)	0.4438 (2)	0.70169 (9)	0.0511 (6)	
C32	0.1542 (7)	0.7098 (6)	0.7540 (3)	0.163 (2)	
H32A	0.0821	0.7381	0.7698	0.244*	
H32B	0.2378	0.7227	0.7726	0.244*	
H32C	0.1429	0.6243	0.7475	0.244*	
C33	0.2519 (5)	0.7433 (6)	0.6824 (2)	0.160 (3)	
H33A	0.2429	0.7970	0.6566	0.241*	
H33B	0.2373	0.6606	0.6723	0.241*	
H33C	0.3403	0.7509	0.6983	0.241*	
C34	0.0645 (5)	0.8613 (4)	0.70234 (18)	0.1072 (14)	
H34	0.0688	0.9030	0.6752	0.129*	
Cl1	0.17980 (9)	0.06784 (7)	0.58331 (3)	0.0737 (2)	
N1	0.7077 (3)	0.2271 (2)	0.59336 (8)	0.0769 (8)	
N2	0.7070 (2)	0.41714 (18)	0.53544 (7)	0.0451 (5)	
N3	0.5667 (3)	0.6108 (2)	0.55944 (8)	0.0645 (6)	
N4	0.4683 (2)	0.3990 (2)	0.65858 (7)	0.0567 (6)	
N5	0.1541 (4)	0.7757 (4)	0.71246 (16)	0.1093 (11)	
O1	0.70259 (19)	0.5143 (2)	0.67959 (6)	0.0649 (5)	
O2	0.3231 (3)	0.0703 (3)	0.58888 (11)	0.1059 (9)	
O3	0.1299 (4)	−0.0427 (3)	0.56646 (13)	0.1260 (12)	
O4	0.1325 (5)	0.0871 (4)	0.62572 (16)	0.180 (2)	
O5	0.1337 (3)	0.1632 (3)	0.55563 (17)	0.1655 (18)	
O6	−0.0233 (4)	0.8916 (3)	0.72468 (14)	0.1380 (13)	

### Atomic displacement parameters (Å^2^)

**Table:**

	*U*^11^	*U*^22^	*U*^33^	*U*^12^	*U*^13^	*U*^23^
Ag1	0.08213 (18)	0.08620 (19)	0.04559 (13)	−0.00483 (12)	0.03491 (11)	−0.00590 (10)
C1	0.160 (4)	0.082 (2)	0.082 (2)	0.015 (3)	0.077 (3)	0.026 (2)
C2	0.158 (4)	0.078 (3)	0.100 (3)	0.021 (3)	0.076 (3)	0.040 (2)
C3	0.107 (3)	0.068 (2)	0.106 (3)	0.0214 (19)	0.055 (2)	0.028 (2)
C4	0.081 (2)	0.0640 (19)	0.0718 (19)	0.0146 (15)	0.0415 (17)	0.0185 (14)
C5	0.0590 (15)	0.0576 (16)	0.0474 (14)	0.0007 (12)	0.0221 (12)	0.0081 (12)
C6	0.0492 (13)	0.0519 (14)	0.0403 (12)	−0.0003 (11)	0.0156 (10)	0.0025 (10)
C7	0.0544 (14)	0.0475 (14)	0.0483 (13)	0.0059 (11)	0.0227 (11)	0.0026 (11)
C8	0.0407 (12)	0.0509 (13)	0.0337 (11)	−0.0011 (10)	0.0109 (9)	−0.0006 (9)
C9	0.0505 (14)	0.0482 (13)	0.0409 (12)	0.0029 (11)	0.0134 (10)	0.0034 (10)
C10	0.0427 (13)	0.0500 (14)	0.0383 (12)	−0.0016 (10)	0.0108 (10)	−0.0037 (10)
C11	0.0426 (13)	0.0541 (14)	0.0464 (13)	−0.0006 (11)	0.0120 (10)	−0.0076 (11)
C12	0.0659 (17)	0.0560 (17)	0.0712 (18)	0.0059 (14)	0.0245 (14)	−0.0010 (14)
C13	0.082 (2)	0.0589 (19)	0.100 (3)	0.0136 (16)	0.0305 (19)	−0.0012 (17)
C14	0.077 (2)	0.074 (2)	0.097 (3)	0.0214 (18)	0.0289 (19)	−0.0159 (19)
C15	0.081 (2)	0.092 (3)	0.076 (2)	0.0163 (19)	0.0429 (17)	−0.0136 (19)
C16	0.0448 (12)	0.0531 (14)	0.0370 (11)	0.0004 (11)	0.0143 (10)	−0.0004 (10)
C17	0.110 (3)	0.0582 (18)	0.078 (2)	0.0189 (17)	0.0595 (19)	0.0140 (15)
C18	0.111 (3)	0.0619 (19)	0.081 (2)	0.0178 (18)	0.062 (2)	0.0014 (16)
C19	0.0544 (15)	0.0674 (17)	0.0453 (13)	−0.0058 (13)	0.0220 (11)	−0.0067 (12)
C20	0.082 (2)	0.0683 (18)	0.0608 (17)	0.0028 (16)	0.0380 (16)	0.0150 (14)
C21	0.0707 (18)	0.0545 (15)	0.0611 (16)	0.0067 (13)	0.0331 (14)	0.0062 (13)
C22	0.0670 (18)	0.084 (2)	0.0498 (15)	−0.0139 (15)	0.0280 (13)	−0.0125 (14)
C23	0.0615 (16)	0.0705 (18)	0.0409 (13)	0.0039 (13)	0.0286 (12)	−0.0040 (12)
C24	0.081 (2)	0.094 (2)	0.0510 (16)	−0.0151 (17)	0.0307 (15)	−0.0187 (15)
C25	0.103 (3)	0.122 (3)	0.0496 (17)	−0.020 (2)	0.0410 (18)	−0.0262 (18)
C26	0.092 (2)	0.103 (3)	0.0559 (17)	−0.009 (2)	0.0482 (17)	−0.0122 (17)
C27	0.0656 (17)	0.0709 (18)	0.0523 (15)	0.0054 (14)	0.0329 (13)	0.0022 (13)
C28	0.070 (2)	0.083 (2)	0.069 (2)	0.0011 (16)	0.0406 (16)	0.0095 (17)
C29	0.0655 (19)	0.089 (2)	0.071 (2)	−0.0088 (17)	0.0203 (15)	0.0065 (17)
C30	0.072 (2)	0.094 (2)	0.0502 (16)	−0.0101 (17)	0.0173 (14)	−0.0025 (15)
C31	0.0598 (16)	0.0558 (15)	0.0426 (13)	0.0086 (12)	0.0269 (12)	0.0015 (10)
C32	0.172 (6)	0.138 (5)	0.174 (6)	0.005 (4)	0.000 (5)	0.008 (5)
C33	0.113 (4)	0.151 (5)	0.228 (7)	0.012 (4)	0.065 (4)	−0.052 (5)
C34	0.103 (3)	0.095 (3)	0.132 (4)	−0.008 (3)	0.045 (3)	−0.008 (3)
Cl1	0.0887 (6)	0.0615 (5)	0.0756 (5)	−0.0031 (4)	0.0286 (4)	−0.0032 (4)
N1	0.108 (2)	0.0706 (17)	0.0612 (15)	0.0056 (15)	0.0482 (15)	0.0132 (12)
N2	0.0458 (11)	0.0533 (12)	0.0384 (10)	−0.0010 (9)	0.0137 (8)	0.0005 (9)
N3	0.0684 (15)	0.0729 (16)	0.0570 (14)	0.0133 (12)	0.0274 (12)	−0.0021 (12)
N4	0.0608 (14)	0.0696 (15)	0.0431 (11)	−0.0014 (11)	0.0212 (10)	0.0008 (10)
N5	0.099 (3)	0.094 (3)	0.138 (3)	−0.003 (2)	0.024 (2)	−0.016 (2)
O1	0.0634 (12)	0.0923 (15)	0.0450 (10)	−0.0149 (10)	0.0314 (9)	−0.0126 (10)
O2	0.0906 (19)	0.113 (2)	0.115 (2)	0.0127 (15)	0.0156 (16)	0.0025 (16)
O3	0.158 (3)	0.0764 (18)	0.151 (3)	−0.0307 (19)	0.051 (2)	−0.0370 (19)
O4	0.185 (4)	0.239 (5)	0.130 (3)	−0.052 (3)	0.081 (3)	−0.081 (3)
O5	0.091 (2)	0.121 (3)	0.284 (5)	0.0051 (19)	0.021 (3)	0.105 (3)
O6	0.125 (3)	0.140 (3)	0.165 (3)	0.011 (2)	0.082 (2)	−0.011 (2)

### Geometric parameters (Å, °)

**Table:**

Ag1—O1	2.5995 (19)	C19—C20	1.370 (4)
Ag1—N1	2.434 (3)	C19—C22	1.508 (3)
Ag1—N2	2.3572 (19)	C20—C21	1.390 (4)
Ag1—N3	2.414 (3)	C20—H20	0.9300
Ag1—N4	2.275 (2)	C21—H21	0.9300
C1—N1	1.338 (4)	C22—O1^i^	1.426 (3)
C1—C2	1.361 (5)	C22—H22A	0.9700
C1—H1	0.9300	C22—H22B	0.9700
C2—C3	1.363 (5)	C23—C24	1.367 (4)
C2—H2	0.9300	C23—O1	1.373 (3)
C3—C4	1.382 (4)	C23—C31	1.418 (4)
C3—H3	0.9300	C24—C25	1.419 (4)
C4—C5	1.377 (4)	C24—H24	0.9300
C4—H4	0.9300	C25—C26	1.347 (5)
C5—N1	1.341 (3)	C25—H25	0.9300
C5—C6	1.489 (3)	C26—C27	1.406 (4)
C6—N2	1.338 (3)	C26—H26	0.9300
C6—C7	1.392 (3)	C27—C28	1.410 (5)
C7—C8	1.392 (3)	C27—C31	1.421 (3)
C7—H7	0.9300	C28—C29	1.348 (5)
C8—C9	1.395 (3)	C28—H28	0.9300
C8—C16	1.491 (3)	C29—C30	1.402 (4)
C9—C10	1.387 (3)	C29—H29	0.9300
C9—H9	0.9300	C30—N4	1.318 (4)
C10—N2	1.335 (3)	C30—H30	0.9300
C10—C11	1.501 (3)	C31—N4	1.371 (3)
C11—N3	1.335 (3)	C32—N5	1.428 (7)
C11—C12	1.378 (4)	C32—H32A	0.9600
C12—C13	1.380 (4)	C32—H32B	0.9600
C12—H12	0.9300	C32—H32C	0.9600
C13—C14	1.372 (5)	C33—N5	1.448 (6)
C13—H13	0.9300	C33—H33A	0.9600
C14—C15	1.359 (5)	C33—H33B	0.9600
C14—H14	0.9300	C33—H33C	0.9600
C15—N3	1.337 (4)	C34—O6	1.211 (5)
C15—H15	0.9300	C34—N5	1.310 (6)
C16—C17	1.374 (4)	C34—H34	0.9300
C16—C21	1.379 (4)	Cl1—O5	1.375 (3)
C17—C18	1.385 (4)	Cl1—O3	1.382 (3)
C17—H17	0.9300	Cl1—O4	1.412 (4)
C18—C19	1.363 (4)	Cl1—O2	1.432 (3)
C18—H18	0.9300	O1—C22^i^	1.426 (4)
			
N4—Ag1—N2	167.56 (8)	O1^i^—C22—C19	107.7 (2)
N4—Ag1—N3	115.30 (8)	O1^i^—C22—H22A	110.2
N2—Ag1—N3	68.62 (7)	C19—C22—H22A	110.2
N4—Ag1—N1	106.16 (8)	O1^i^—C22—H22B	110.2
N2—Ag1—N1	68.55 (7)	C19—C22—H22B	110.2
N3—Ag1—N1	137.12 (7)	H22A—C22—H22B	108.5
N4—Ag1—O1	66.35 (7)	C24—C23—O1	124.4 (3)
N2—Ag1—O1	125.64 (6)	C24—C23—C31	120.9 (2)
N3—Ag1—O1	98.95 (8)	O1—C23—C31	114.7 (2)
N1—Ag1—O1	107.45 (9)	C23—C24—C25	119.5 (3)
N1—C1—C2	123.4 (3)	C23—C24—H24	120.3
N1—C1—H1	118.3	C25—C24—H24	120.3
C2—C1—H1	118.3	C26—C25—C24	121.1 (3)
C1—C2—C3	118.9 (3)	C26—C25—H25	119.5
C1—C2—H2	120.5	C24—C25—H25	119.5
C3—C2—H2	120.5	C25—C26—C27	120.7 (3)
C2—C3—C4	118.7 (3)	C25—C26—H26	119.7
C2—C3—H3	120.7	C27—C26—H26	119.7
C4—C3—H3	120.7	C26—C27—C28	122.9 (3)
C5—C4—C3	119.6 (3)	C26—C27—C31	119.5 (3)
C5—C4—H4	120.2	C28—C27—C31	117.6 (3)
C3—C4—H4	120.2	C29—C28—C27	119.9 (3)
N1—C5—C4	121.4 (2)	C29—C28—H28	120.1
N1—C5—C6	116.3 (2)	C27—C28—H28	120.1
C4—C5—C6	122.3 (2)	C28—C29—C30	119.2 (3)
N2—C6—C7	121.6 (2)	C28—C29—H29	120.4
N2—C6—C5	116.5 (2)	C30—C29—H29	120.4
C7—C6—C5	121.8 (2)	N4—C30—C29	123.6 (3)
C6—C7—C8	120.1 (2)	N4—C30—H30	118.2
C6—C7—H7	120.0	C29—C30—H30	118.2
C8—C7—H7	120.0	N4—C31—C23	120.2 (2)
C7—C8—C9	117.0 (2)	N4—C31—C27	121.5 (3)
C7—C8—C16	121.3 (2)	C23—C31—C27	118.3 (2)
C9—C8—C16	121.7 (2)	N5—C32—H32A	109.5
C10—C9—C8	120.1 (2)	N5—C32—H32B	109.5
C10—C9—H9	119.9	H32A—C32—H32B	109.5
C8—C9—H9	119.9	N5—C32—H32C	109.5
N2—C10—C9	121.8 (2)	H32A—C32—H32C	109.5
N2—C10—C11	116.2 (2)	H32B—C32—H32C	109.5
C9—C10—C11	122.0 (2)	N5—C33—H33A	109.5
N3—C11—C12	121.5 (2)	N5—C33—H33B	109.5
N3—C11—C10	116.5 (2)	H33A—C33—H33B	109.5
C12—C11—C10	122.0 (2)	N5—C33—H33C	109.5
C11—C12—C13	119.5 (3)	H33A—C33—H33C	109.5
C11—C12—H12	120.2	H33B—C33—H33C	109.5
C13—C12—H12	120.2	O6—C34—N5	126.6 (5)
C14—C13—C12	119.1 (3)	O6—C34—H34	116.7
C14—C13—H13	120.5	N5—C34—H34	116.7
C12—C13—H13	120.5	O5—Cl1—O3	111.6 (3)
C15—C14—C13	117.8 (3)	O5—Cl1—O4	107.0 (3)
C15—C14—H14	121.1	O3—Cl1—O4	107.9 (2)
C13—C14—H14	121.1	O5—Cl1—O2	108.57 (19)
N3—C15—C14	124.4 (3)	O3—Cl1—O2	112.1 (2)
N3—C15—H15	117.8	O4—Cl1—O2	109.6 (2)
C14—C15—H15	117.8	C1—N1—C5	117.9 (3)
C17—C16—C21	117.1 (2)	C1—N1—Ag1	123.7 (2)
C17—C16—C8	120.9 (2)	C5—N1—Ag1	117.28 (18)
C21—C16—C8	122.0 (2)	C10—N2—C6	119.36 (19)
C16—C17—C18	121.3 (3)	C10—N2—Ag1	119.76 (15)
C16—C17—H17	119.3	C6—N2—Ag1	119.77 (15)
C18—C17—H17	119.3	C11—N3—C15	117.7 (3)
C19—C18—C17	121.6 (3)	C11—N3—Ag1	117.99 (18)
C19—C18—H18	119.2	C15—N3—Ag1	124.3 (2)
C17—C18—H18	119.2	C30—N4—C31	118.2 (2)
C18—C19—C20	117.6 (2)	C30—N4—Ag1	118.02 (19)
C18—C19—C22	121.2 (3)	C31—N4—Ag1	123.75 (18)
C20—C19—C22	121.2 (3)	C34—N5—C32	119.4 (5)
C19—C20—C21	121.2 (3)	C34—N5—C33	122.2 (5)
C19—C20—H20	119.4	C32—N5—C33	118.4 (5)
C21—C20—H20	119.4	C23—O1—C22^i^	117.5 (2)
C16—C21—C20	121.1 (3)	C23—O1—Ag1	114.95 (16)
C16—C21—H21	119.5	C22^i^—O1—Ag1	127.44 (14)
C20—C21—H21	119.5		
			
N1—C1—C2—C3	−2.1 (8)	N4—Ag1—N1—C1	−1.9 (4)
C1—C2—C3—C4	0.6 (7)	N2—Ag1—N1—C1	−170.0 (4)
C2—C3—C4—C5	0.8 (6)	N3—Ag1—N1—C1	−166.9 (3)
C3—C4—C5—N1	−1.0 (5)	O1—Ag1—N1—C1	67.8 (4)
C3—C4—C5—C6	−178.9 (3)	N4—Ag1—N1—C5	165.8 (2)
N1—C5—C6—N2	−14.4 (4)	N2—Ag1—N1—C5	−2.3 (2)
C4—C5—C6—N2	163.7 (3)	N3—Ag1—N1—C5	0.9 (3)
N1—C5—C6—C7	166.4 (3)	O1—Ag1—N1—C5	−124.5 (2)
C4—C5—C6—C7	−15.5 (4)	C9—C10—N2—C6	0.5 (4)
N2—C6—C7—C8	−0.8 (4)	C11—C10—N2—C6	−179.2 (2)
C5—C6—C7—C8	178.4 (2)	C9—C10—N2—Ag1	168.45 (18)
C6—C7—C8—C9	1.4 (4)	C11—C10—N2—Ag1	−11.2 (3)
C6—C7—C8—C16	−177.6 (2)	C7—C6—N2—C10	−0.2 (4)
C7—C8—C9—C10	−1.1 (4)	C5—C6—N2—C10	−179.4 (2)
C16—C8—C9—C10	177.9 (2)	C7—C6—N2—Ag1	−168.13 (19)
C8—C9—C10—N2	0.2 (4)	C5—C6—N2—Ag1	12.7 (3)
C8—C9—C10—C11	179.8 (2)	N4—Ag1—N2—C10	119.3 (4)
N2—C10—C11—N3	6.6 (3)	N3—Ag1—N2—C10	8.49 (18)
C9—C10—C11—N3	−173.0 (2)	N1—Ag1—N2—C10	−173.8 (2)
N2—C10—C11—C12	−171.3 (2)	O1—Ag1—N2—C10	−77.2 (2)
C9—C10—C11—C12	9.0 (4)	N4—Ag1—N2—C6	−72.7 (4)
N3—C11—C12—C13	2.1 (5)	N3—Ag1—N2—C6	176.4 (2)
C10—C11—C12—C13	180.0 (3)	N1—Ag1—N2—C6	−5.86 (18)
C11—C12—C13—C14	−0.8 (5)	O1—Ag1—N2—C6	90.73 (19)
C12—C13—C14—C15	−0.5 (6)	C12—C11—N3—C15	−1.9 (4)
C13—C14—C15—N3	0.7 (6)	C10—C11—N3—C15	−179.9 (3)
C7—C8—C16—C17	8.0 (4)	C12—C11—N3—Ag1	178.9 (2)
C9—C8—C16—C17	−170.9 (3)	C10—C11—N3—Ag1	0.9 (3)
C7—C8—C16—C21	−172.5 (3)	C14—C15—N3—C11	0.5 (5)
C9—C8—C16—C21	8.6 (4)	C14—C15—N3—Ag1	179.7 (3)
C21—C16—C17—C18	−2.6 (5)	N4—Ag1—N3—C11	−171.7 (2)
C8—C16—C17—C18	176.9 (3)	N2—Ag1—N3—C11	−4.6 (2)
C16—C17—C18—C19	−1.3 (6)	N1—Ag1—N3—C11	−7.7 (3)
C17—C18—C19—C20	3.7 (6)	O1—Ag1—N3—C11	120.3 (2)
C17—C18—C19—C22	−174.7 (3)	N4—Ag1—N3—C15	9.1 (3)
C18—C19—C20—C21	−2.3 (5)	N2—Ag1—N3—C15	176.3 (3)
C22—C19—C20—C21	176.2 (3)	N1—Ag1—N3—C15	173.1 (3)
C17—C16—C21—C20	4.0 (5)	O1—Ag1—N3—C15	−58.9 (3)
C8—C16—C21—C20	−175.5 (3)	C29—C30—N4—C31	1.5 (5)
C19—C20—C21—C16	−1.6 (5)	C29—C30—N4—Ag1	−176.6 (3)
C18—C19—C22—O1^i^	−109.7 (3)	C23—C31—N4—C30	178.8 (3)
C20—C19—C22—O1^i^	72.0 (4)	C27—C31—N4—C30	−0.4 (4)
O1—C23—C24—C25	−179.4 (3)	C23—C31—N4—Ag1	−3.3 (3)
C31—C23—C24—C25	1.1 (5)	C27—C31—N4—Ag1	177.55 (19)
C23—C24—C25—C26	−1.5 (6)	N2—Ag1—N4—C30	−14.0 (5)
C24—C25—C26—C27	1.1 (6)	N3—Ag1—N4—C30	91.7 (2)
C25—C26—C27—C28	−179.8 (4)	N1—Ag1—N4—C30	−77.0 (2)
C25—C26—C27—C31	−0.2 (5)	O1—Ag1—N4—C30	−179.3 (3)
C26—C27—C28—C29	−178.3 (3)	N2—Ag1—N4—C31	168.1 (3)
C31—C27—C28—C29	2.1 (5)	N3—Ag1—N4—C31	−86.2 (2)
C27—C28—C29—C30	−1.2 (5)	N1—Ag1—N4—C31	105.0 (2)
C28—C29—C30—N4	−0.7 (6)	O1—Ag1—N4—C31	2.70 (19)
C24—C23—C31—N4	−179.5 (3)	O6—C34—N5—C32	−1.1 (8)
O1—C23—C31—N4	1.0 (4)	O6—C34—N5—C33	177.5 (5)
C24—C23—C31—C27	−0.3 (4)	C24—C23—O1—C22^i^	−1.6 (4)
O1—C23—C31—C27	−179.8 (2)	C31—C23—O1—C22^i^	178.0 (3)
C26—C27—C31—N4	179.0 (3)	C24—C23—O1—Ag1	−178.2 (3)
C28—C27—C31—N4	−1.3 (4)	C31—C23—O1—Ag1	1.3 (3)
C26—C27—C31—C23	−0.2 (4)	N4—Ag1—O1—C23	−2.03 (18)
C28—C27—C31—C23	179.4 (3)	N2—Ag1—O1—C23	−178.19 (17)
C2—C1—N1—C5	2.0 (7)	N3—Ag1—O1—C23	111.75 (19)
C2—C1—N1—Ag1	169.7 (4)	N1—Ag1—O1—C23	−102.4 (2)
C4—C5—N1—C1	−0.4 (5)	N4—Ag1—O1—C22^i^	−178.3 (3)
C6—C5—N1—C1	177.7 (3)	N2—Ag1—O1—C22^i^	5.5 (3)
C4—C5—N1—Ag1	−168.9 (2)	N3—Ag1—O1—C22^i^	−64.5 (2)
C6—C5—N1—Ag1	9.2 (3)	N1—Ag1—O1—C22^i^	81.3 (3)

Symmetry codes: (i) −*x*+2, −*y*+1, −*z*+1.

### Hydrogen-bond geometry (Å, °)

**Table:**

*D*—H···*A*	*D*—H	H···*A*	*D*···*A*	*D*—H···*A*
C4—H4···O3^ii^	0.93	2.57	3.450 (5)	158
C12—H12···O5^iii^	0.93	2.60	3.344 (5)	138
C28—H28···O6^iv^	0.93	2.37	3.250 (5)	158
C29—H29···O4	0.93	2.57	3.252 (6)	130

Symmetry codes: (ii) −*x*+1, −*y*, −*z*+1; (iii) −*x*+1, −*y*+1, −*z*+1; (iv) −*x*, *y*−1/2, −*z*+3/2.
